# Dataset on the thermal performance of green roofs and green walls in a temperate continental climate: An experimental approach in Quito, Ecuador

**DOI:** 10.1016/j.dib.2025.111652

**Published:** 2025-05-15

**Authors:** Marcelo Villacis-Ormaza

**Affiliations:** Faculty of Architecture, Design and Art, Universidad Tecnológica Indoamérica, Quito 170103, Ecuador

**Keywords:** Green roof, Green wall, Relative humidity, Living wall, Thermal performance

## Abstract

This dataset comprises temperature and humidity measurements obtained from two experimental building prototypes designed to characterize the thermal behavior of green roofs and green walls in a temperate continental climate. The data was collected at an elevation of 2850 m above sea level in Quito, Ecuador (−0.120694, −78.498677). At 15-min intervals, calibrated digital sensors recorded the temperature and relative humidity indoors over the course of four months. Values from two configurations are included in the dataset: one that integrates green infrastructure and another that acts as a base case devoid of vegetation. With possible uses for building simulations, urban climate studies, and policy creation, the data are organized to promote study on thermal comfort, energy efficiency, and sustainable construction methods.

Specifications Table

**Table:** 

Subject	Engineering & Materials science
Specific subject area	*Thermal performance of green roofs and walls in high-altitude, temperate continental climates.*
Type of data	Tables .xlsx
Data collection	*Digital sensors (±0.5 °C accuracy, ±3 % RH) with data logging managed using Elite Logwin software*
Data source location	*Quito, Ecuador (−0.120694, −78.498677), Quito 170103*
Data accessibility	Repository name: Mendeley Data Data identification number: 10.17632/y77xtf7w34.1 Direct URL to data: https://data.mendeley.com/datasets/y77xtf7w34/1
Related research article	An Experimental Study on the Thermal Performance of Intensive Green Walls and Green Roofs in Temperate Continental Climatic Zones. Pre-print

## 1 Value of the Data

• Supports the evaluation of green roofs and walls in non-tropical high-altitude climates.
• Helps validate thermal performance models for sustainable building designs.
• Facilitates comparative studies between green infrastructure in different climatic zones.
• Can be used by policymakers to develop building regulations promoting energy efficiency.
• Enables integration into urban climate models for improved city planning.
• Serves as a reference dataset for future experimental studies on passive cooling techniques

## 2 Background

**Fig. 1:**
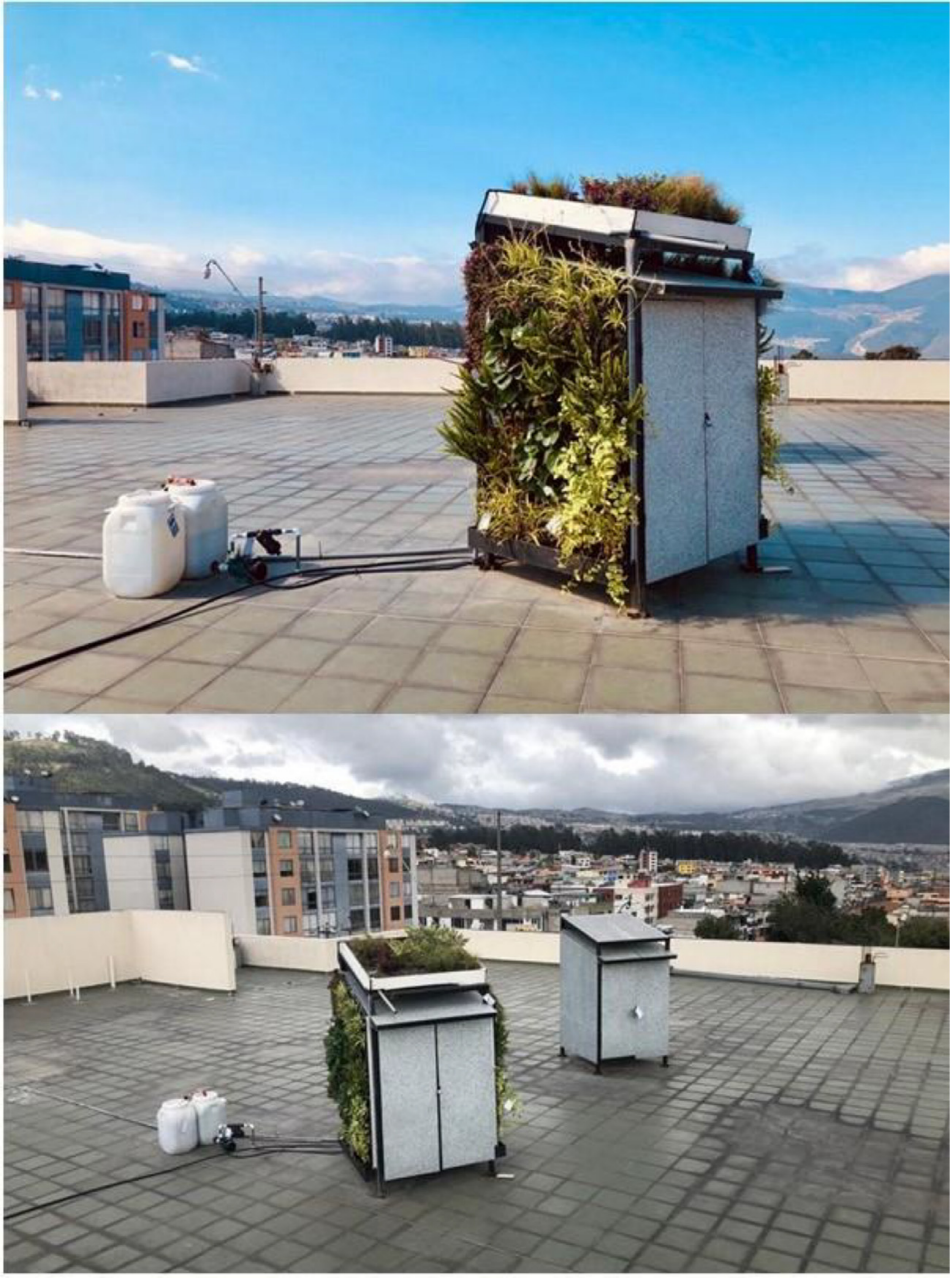
Images of base case prototype and vegetated prototype.

The increasing interest in sustainable building techniques and urban resilience tactics in high-altitude settings is driven by the need to address energy efficiency and thermal comfort in regions with unique climate challenges. Vegetated walls and roofs are examples of green infrastructure that has been extensively researched in temperate and tropical countries, but it is still little understood in cities with a variety of climatic challenges, such as Quito (ASHRAE Climate Zone 4C) [[1], [2], [3]]. Recent studies highlight the potential of green roofs to reduce surface temperatures by up to 5 °C in Mediterranean climates [4] and green walls to lower internal mean temperatures by 2.2 °C and maximum reduction up to 19.8 °C in tropical zones [5], but comparable data for high-altitude regions is sparse (Fig. 1).

Recent research on green infrastructure has highlighted its effectiveness in enhancing thermal performance across various climatic conditions. Studies conducted in high-altitude regions have demonstrated that green roofs and green walls contribute to mitigating urban heat island effects and improving indoor thermal comfort by reducing temperature fluctuations [6]. In temperate continental climates, vegetation-based systems have been shown to provide insulation benefits, particularly during extreme seasonal variations [7]. However, the specific thermal behavior of green infrastructure in high-altitude environments, such as Quito, remains underexplored. Comparative analyses between different climate zones suggest that factors such as altitude, solar radiation intensity, and humidity levels significantly influence the thermal performance of vegetated surfaces [8]. Recent studies from 2020 to 2025 have further examined the role of green infrastructure in energy efficiency, emphasizing the need for localized experimental assessments to validate broader theoretical models [9].

Additionally, advancements in sustainable construction techniques have underscored the importance of integrating vegetation-based solutions to enhance building stability under fluctuating thermal conditions. Research on thermal stability in buildings has indicated that vegetative layers not only reduce external temperature impacts but also regulate internal humidity levels, contributing to long-term structural resilience. The interaction between substrate composition, plant species, and insulation properties has been widely studied, with findings suggesting that optimized green roof and wall configurations can enhance thermal inertia and reduce energy demands. Recent investigations have also explored the combined effects of green infrastructure and passive cooling strategies, reinforcing the necessity of empirical studies to validate simulation-based findings. By incorporating recent literature on these topics, the present study aims to address the existing knowledge gap regarding the specific thermal dynamics of green roofs and walls in high-altitude urban contexts.

This dataset attempts to offer empirical proof of the thermal regulation capabilities of green walls and roofs by methodically gathering temperature and relative humidity data. The dataset supports current studies on passive design techniques and urban heat reduction, aiding in the creation of regional building standards and energy-efficient architectural solutions. The dataset also aligns with global efforts to expand climate-responsive architectural solutions, as highlighted in the 2023 IPCC report on urban sustainability [10].

## 3 Data Description

The dataset [11] is organized into two main categories: processed data and figures. The processed data consists of temperature and relative humidity readings from two prototypes, one equipped with green walls and a modular green roof (Prototype 1 - Vegetated), and the other serving as a non-vegetated baseline (Prototype 2 - Non-Vegetated). Data was collected during four different seasonal periods: July 4-8, 2022, July 11-19, 2022, July 25 - August 8, 2022, and August 19 - September 22, 2022. The readings are taken every 15 minutes and include internal temperature (°C) as well as relative humidity (%) for each prototype. In addition, the dataset includes the Thermal Comfort standard values in Celsius degrees, according to the Ecuadorian Building Code (Norma Ecuatoriana de la Construcción, NEC [12]), to assess how each prototype meets comfort standards during each time period. The data is presented in Excel files (.xls), which include both the time-stamped readings and the processed statistical summaries [11].

The processed data is derived from the raw readings and provides statistical summaries, such as mean values, standard deviations, and thermal performance comparisons between the vegetated and non-vegetated prototypes. These summaries help to identify trends in temperature and relative humidity fluctuations across dry and rainy periods, and to evaluate the effectiveness of the green infrastructure in moderating indoor environmental conditions. This processed data is also essential for comparing the two prototypes in terms of thermal comfort and energy efficiency, enabling a more comprehensive understanding of how green roofs and walls perform in temperate continental climates like that of Quito.

**Fig. 2:**
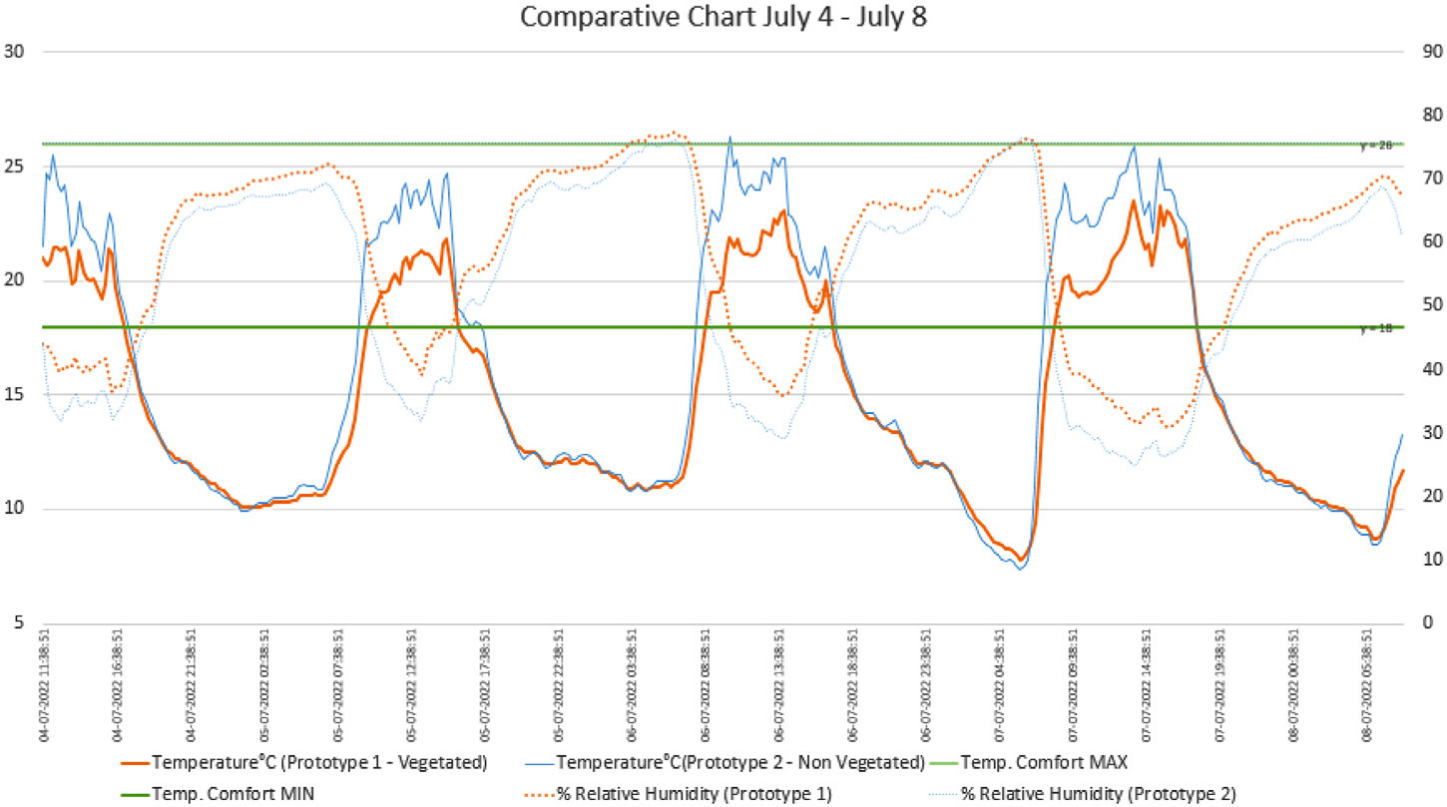
Comparison chart of base case prototype and vegetated prototype. July 4–8.

**Fig. 3:**
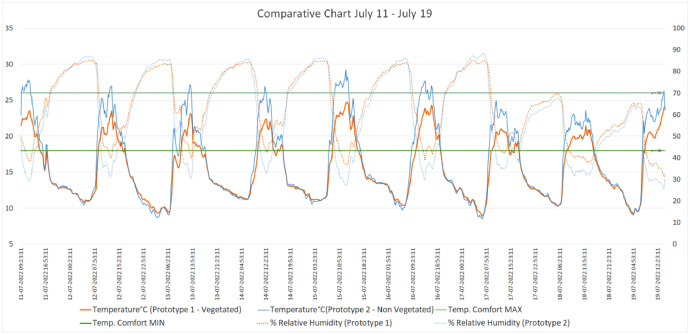
Comparison chart of base case prototype and vegetated prototype. July11–19.

**Fig. 4:**
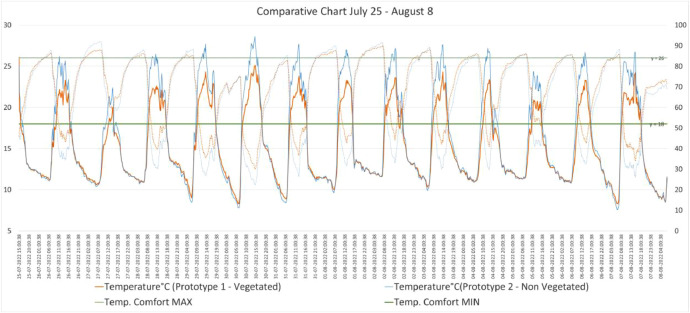
Comparison chart of base case prototype and vegetated prototype. July25–Aug19.

The figures section is integrated into the dataset itself, and contains various graphs that illustrate temperature fluctuations, humidity variations, and performance differentials between the two prototypes. The graphs are designed to provide a clear visual representation of the data, allowing for easy comparison between the vegetated and non-vegetated configurations. These figures include temperature and humidity profiles for each prototype during the specified study periods and also highlight how each configuration aligns with the thermal comfort standards as noted in Fig. 2, Fig. 3, Fig. 4. Each graph is accompanied by captions that help interpret the data, though further analysis or conclusions are not included, leaving that open for future interpretation.

Additional visualizations are available within the dataset itself, providing further insights into the recorded temperature variations. These supplementary graphical analyses can be accessed for a more detailed examination of the findings.

## 4 Experimental Design, Materials and Methods

The experimental prototype approach was selected over alternative methods, such as simulation models or field studies in existing buildings, due to its ability to provide controlled conditions for assessing the thermal performance of green roofs and walls. Unlike simulations, which rely on assumptions and predefined parameters that may not fully capture real-world complexities, physical prototypes enable direct measurement of temperature and humidity variations in a controlled environment, ensuring that the impact of vegetation can be isolated more effectively. Additionally, field studies in existing buildings introduce multiple confounding factors, such as variations in construction materials, occupancy patterns, and microclimatic influences, which can obscure the specific effects of green infrastructure. By employing experimental prototypes, this study minimizes external variability and enhances the reliability of the recorded data, thereby providing a robust foundation for evaluating the thermal behavior of green roofs and walls in high-altitude, temperate continental climates.

In this study, descriptive statistical methods were employed to analyze temperature and humidity variations over time. The dataset, consisting of continuous time-series measurements, was summarized using mean and standard deviation to identify overall trends in thermal performance. Given the observational nature of the study, emphasis was placed on descriptive rather than inferential statistical analyses. Normality tests, such as the Shapiro–Wilk test, were not performed, as the primary objective was to characterize general trends rather than conduct hypothesis testing.

Since this study was based on experimental prototypes rather than a traditional population sample, a conventional sample size calculation was not applicable. Instead, the duration of data collection and the frequency of measurements were carefully designed to ensure sufficient representation of thermal behavior across different environmental conditions. Data were recorded over a period of four months, which allowed seasonal variations and daily thermal fluctuations to be captured effectively. Measurements were taken at 15-min intervals, ensuring a high-resolution dataset capable of reflecting short-term and long-term temperature dynamics.

The chosen timeframe was deemed adequate for capturing diurnal and nocturnal temperature variations, as well as potential thermal lag effects associated with vegetated surfaces.

The data was collected from two experimental prototypes [13], both measuring 1.44 m² in surface area and 2.88 m³ in volume, constructed using recycled Tetra Pak polyaluminum panels. These panels were chosen for their sustainable properties, providing a stable, durable structure for the prototypes. One prototype was equipped with hydroponic green walls and a modular 800 mm soil substrate green roof to represent the vegetated infrastructure (Prototype 1) (Table1), while the second prototype was left as a baseline with no vegetation (Prototype 2). The aim was to compare the thermal performance of the vegetated prototype against the non-vegetated baseline to assess how green infrastructure influences indoor temperature and humidity levels in a temperate continental climate.

Temperature and relative humidity data were recorded using calibrated digital sensors, which were strategically placed inside and outside both prototypes. The sensors were set to automatically record data at 15-minute intervals, allowing for continuous monitoring over distinct seasonal periods: July 4–8, 2022, July 11–19, 2022, July 25 - August 8, 2022, and August 19–September 22, 2022. The data was logged and stored in Excel files, which include detailed timestamped readings for each of the variables. The study was designed to capture seasonal variations, ensuring a comprehensive dataset that spans different weather conditions and evaluates the performance of the prototypes in both warmer and cooler conditions.

The sensors were calibrated to provide high accuracy, with temperature measurements recorded to ±0.5 °C and relative humidity to ±3 %. The dataset also includes thermal comfort standards based on the Norma Ecuatoriana de la Construcción (NEC) [12], which were used to assess whether the thermal conditions inside each prototype met the recommended comfort levels (Table 1). The data was analyzed in terms of mean values, standard deviations, and overall performance comparisons. The software used for logging and organizing the data was Elite Logwin, which facilitated easy storage and processing of the information. This setup ensured that the data collection was both precise and consistent, providing a robust dataset for evaluating the thermal performance of green infrastructure in Quito’s high-altitude climate.

**Table 1:** Plants list for vegetated prototype.

No.	Scientific name	Common name	Quantity of plants Green Roof	Quantity of plants Green Wall
1	Alternanthera porrigens	Alcancel Moradilla	4	18
2	Chlorophytum comosum	Mala Madre	13	38
3	Nephrolepis exaltata	Helecho	4	27
4	Magnoliopsida	Vinca	4	39
5	Bergenia crassifolia	Hortensia	4	23
6	Calamagrostis effusa	Pajonal	7	17
7	Aerva Sanguinolentaa	Escancel Rojo	1	20

Furthermore, data consistency was ensured by verifying measurement continuity and removing erroneous values caused by sensor malfunctions. Since parametric statistical tests typically require normality assumptions, their application was not considered necessary for this study (Table 2).

**Table 2:** Characteristics of the measurement instruments.

Parameters	Instrument	Measuring Range	Accuracy
Indoor air temperature (°C)	Elitech RC-4HC	−40 °C to 85 °C	±0,5 °C
Indoor relative humidity (%)	Elitech RC-4HC	0 % to 99 %	±3%RH (25 °C,20%RH to 90 %RH), ±5 %RH(other range)

## Limitations

The primary limitation of this dataset is the duration of data collection, which spans four months. While seasonal variations are partially captured, a longer monitoring period would provide a more comprehensive representation of annual climatic fluctuations.

Sensitivity analyses were not performed in this study, as the experimental design was primarily observational and focused on direct measurements of thermal performance. However, the selection of key parameters, such as sensor placement, plant species, and substrate composition, was informed by prior studies to ensure methodological consistency.

Data quality was ensured through continuous monitoring and validation of recorded measurements. Any missing data points resulting from sensor malfunctions or temporary connectivity issues were identified and excluded from the analysis to maintain dataset integrity.

Despite this, the dataset remains a valuable resource for thermal comfort calculations, offering insights into temperature and humidity variations in vegetated and non-vegetated structures. The recorded data can be used to assess thermal performance and inform further studies on sustainable building strategies.

## Ethics Statement

The authors confirm that they have read and comply with the ethical requirements for publication in Data in Brief. This work does not involve human subjects, animal experiments, or any data collected from social media platforms.

## CRediT Author Statement

**Marcelo Villacis-Ormaza:** Methodology, Data Curation, Visualization, Writing.

## Data Availability

Mendeley DataDataset on the Thermal Performance of Green Roofs and Green Walls in a Temperate Continental Climate (Original data)
